# The diagnostic significance of the ZNF gene family in pancreatic cancer: a bioinformatics and experimental study

**DOI:** 10.3389/fgene.2023.1089023

**Published:** 2023-06-16

**Authors:** Lei Zhu, Dong Tu, Ruixue Li, Lin Li, Wenjie Zhang, Wenxiang Jin, Tiehan Li, Hong Zhu

**Affiliations:** ^1^Department of Hepatobiliary and Pancreatic Surgery, Second Affiliated Hospital of Kunming Medical University, Kunming, Yunnan, China; ^2^Department of Cardiothoracic Surgery, No. 920 Hospital of the PLA Joint Logistics Support Force, Kunming, China

**Keywords:** pancreatic adenocarcinoma, zinc finger protein family, prognostic risk model, TCGA, bioinformatics

## Abstract

**Background:** Pancreatic adenocarcinoma (PAAD) is among the most devastating of all cancers with a poor survival rate. Therefore, we established a zinc finger (ZNF) protein-based prognostic prediction model for PAAD patients.

**Methods:** The RNA–seq data for PAAD were downloaded from The Cancer Genome Atlas (TCGA) and the Gene Expression Omnibus (GEO) databases. Differentially expressed ZNF protein genes (DE-ZNFs) in PAAD and normal control tissues were screened using the “lemma” package in R. An optimal risk model and an independent prognostic value were established by univariate and multivariate Cox regression analyses. Survival analyses were performed to assess the prognostic ability of the model.

**Results:** We constructed a ZNF family genes-related risk score model that is based on the 10 DE-ZNFs (ZNF185, PRKCI, RTP4, SERTAD2, DEF8, ZMAT1, SP110, U2AF1L4, CXXC1, and RMND5B). The risk score was found to be a significant independent prognostic factor for PAAD patients. Seven significantly differentially expressed immune cells were identified between the high- and low-risk patients. Then, based on the prognostic genes, we constructed a ceRNA regulatory network that includes 5 prognostic genes, 7 miRNAs and 35 lncRNAs. Expression analysis showed ZNF185, PRKCI and RTP4 were significantly upregulated, while ZMAT1 and CXXC1 were significantly downregulated in the PAAD samples in all TCGA - PAAD, GSE28735 and GSE15471 datasets. Moreover, the upregulation of RTP4, SERTAD2, and SP110 were verified by the cell experiments.

**Conclusion:** We established and validated a novel, Zinc finger protein family - related prognostic risk model for patients with PAAD, that has the potential to inform patient management.

## 1 Introduction

Pancreatic adenocarcinoma (PAAD) is a devastating malignancy with a very low 5-year survival rate (Kuninty et al., 2019). The onset of PAAD is insidious, and most patients are admitted to the hospital with clinical symptoms such as “jaundice and abdominal pain”. Nearly 80% of patients have no chance of surgical resection (Mizrahi et al., 2020). Currently, the main diagnostic option for PAAD is imaging. Compared with other mainstream targeted therapies for malignant tumors, PAAD lacks effective target diagnosis and precise treatment options (Grossberg et al., 2020). The pathogenesis of PAAD is regulated by multiple factors, multiple genes and microenvironments. Therefore, the use of massively parallel sequencing technologies to mine PAAD datasets, and utilization of bioinformatics methods to accurately analyze interaction targets, can provide new ideas for precise treatment of PAAD.

The ZNF domain is present in about 5% of human proteins and is associated with pathogenesis of many solid tumors. Precisely, ZNF259 activates ERK/GSK3β by activating the ERK/GSK3β/Snail signaling pathway to promote breast cancer cell invasion and migration (Xiao et al., 2014; Liu et al., 2018). Due to multifunctional binding abilities of zinc finger proteins, ZNF plays an important role in cell differentiation, cell metabolism, autophagy, apoptosis, and stemness maintenance. There is a need to elucidate on the significance of zinc finger proteins in tumor pathogenesis. Through bioinformatics analyses, Sun et al. (2021) found that the zinc finger protein 2 gene (ZIC2) is positively correlated with immune infiltrating cells in hepatocellular carcinoma (HCC) patients, and elevated ZIC2 mRNA expressions in CD4^+^ T cells are associated with the 5-year survival rate of HCC patients. These findings, imply that the ZIC2 gene can be used as a marker for liver cancer immune responses, and to predict HCC prognosis. In pancreatic ductal adenocarcinoma, ZNF185 and SERTAD2 are tumor immune targets, providing new ideas for treatment of tumor immune invasion (Chen et al., 2021; Zhang et al., 2022).

In summary, PAAD pathogenesis is regulated by multiple factors, multiple genes and microenvironments. The ZNF gene may be involved in cancer occurrence and progression as an oncogene. Therefore, the use of massively parallel sequencing technologies to mine PAAD datasets, and utilization of bioinformatics methods to accurately analyze interaction targets, can provide new ideas for precise treatment of PAAD. We mined TCGA, GEO, ICGC, as well as Uniprot databases, and used GSE as the training set to construct a PAAD-related risk score model via COX regression analysis. The constructed risk model is of great significance for further studies on the roles of ZNF in pancreatic cancer pathogenesis.

## 2 Methods

### 2.1 Data sources

The RNA-seq data and corresponding clinical data for TCGA-PAAD patients were downloaded from-the Genomic Data Commons (GDC) database (GDC Data Portal, RRID:SCR_014514) (https://portal.gdc.cancer.gov), which included 178 PAAD cases and 4 control samples. The RNA-seq data of 167 and eight healthy pancreatic tissues samples were obtained from the GTEx and ANTE databases, respectively. The RNA-seq data and survival information for PAAD patients from two countries (PACA-AU: 455 cases; PACA-CA: 455 cases) were downloaded from the International Cancer Genome Consortium (ICGC) database (ICGC Data Portal, RRID:SCR_021722) (https://dcc.icgc.org/). Three PAAD-related datasets (GSE62452, GSE15471, and GSE28735) were downloaded from the Gene Expression Omnibus (GEO) database- (GEO, RRID:SCR_005012) (https://www.ncbi.nlm.nih.gov/geo/). The ICGC and GSE62452 datasets were used as external validation datasets for the prognostic model, and the GSE15471, GSE28735 datasets, as well as the ANTE-normal cohorts, were used to validate the expression levels of the prognostic model genes. The 1986 zinc finger protein family genes were extracted from the universal Protein Resource UniProt database (universal Protein Resource, RRID:SCR_002380) UniProt database (https://www.uniprot.org/) (The UniProt Consortium, 2017).

### 2.2 Analysis of differentially expressed ZNF protein family genes in PAAD

According to the previous literature, the differential analysis was performed between 178 PAAD samples from the TCGA database and 171 normal samples from the GTEx and TCGA databases using the “limma”package (limma, RRID:SCR_010943) (http://bioinf.wehi.edu.au/limma/) in R (Ritchie et al., 2015; Wen et al., 2020). The principal component analysis (PCA) plot in the TCGA-PAAD and GTEx-normal cohorts between the case and normal samples were displayed in Supplementary Figure S1, indicating an excellent distinction. In which, genes with adjusted p-value (adj. p) < 0.05 and-|log2 (fold change)|>1 were considered significantly expressed (Wang et al., 2022). Intersections of differentially expressed genes in PAAD and 1936 PAAD-related ZNF family genes in the UniProt database were used as differentially expressed ZNF family genes (DE-ZNFs). Results were visualized using a heatmap and a volcano plot.

### 2.3 Functional enrichment analysis

Gene Ontology (GO) and Kyoto Encyclopedia of Genes and Genomes (KEGG) enrichment analyses were performed on the DE-ZNFs using clusterProfiler (ClusterProfiler, RRID:SCR_016884) (http://bioconductor.org/packages/release/bioc/html/clusterProfiler.html) (Yu et al., 2012). The GO system consists of three components: biological process (BP), molecular functions (MF) and cellular components (CC). KEGG (KEGG, RRID:SCR_012773) (https://www.kegg.jp) (http://www.genome.jp/kegg/-) is a biological systems database that integrates genomic, chemical and systemic functional information (Kanehisa et al., 2008). The enrichment results were visualized by withusing the “ggplot2” package (ggplot2, RRID:SCR_014601) (https://cran.r-project.org/web/packages/ggplot2/index.html).

### 2.4 Construction of the prognostic signature

Using TCGA as the training set, univariate Cox proportional hazards regression analysis was performed on DE-ZNFs to screen for the genes that were significantly associated with overall survival (OS) outcomes in the TCGA-PAAD training set (*p* < 0.01). Then, patients were divided into high and low expression groups according to expressions of DE-ZNFs from univariate Cox analysis for KM survival analysis to obtain the DE-ZNF with significant different survival rate between the 2 expression groups (*p* < 0.01). These DE-ZNFs were subjected to multivariate Cox regression analyses to identify suitable ZNF - related genes to construct the model. Model genes were screened to calculate the risk scores. A prognostic risk score was calculated for each patient using the formula: risk score = β1 X1 + β2 X2 + …… + βn Xn, whereby β represents the coefficient, X represents prognostic gene expressions and n represents the number of genes. The median risk score was used as the cut-off value to divide the TCGA-PAAD patients into the high and low-risk group. Then, the K-M survival curve was constructed after which, the log-rank test was used to assess survival differences between the risk groups. Sensitivity and specificity of the prognostic model were assessed by ROC analysis and AUC values indicated discrimination. Effectiveness of the prognostic model was validated using the ICGC and GSE63452 datasets.

### 2.5 Survival and risk score analyses

Stratified analysis was performed to establish the correlations between high and low risk groups and survival outcomes in patients with different clinico-pathological characteristics (age >65, age ≤ 65, female, male, M0, T3-T4, stage I-stage II, stage III-stage IⅤ; G1/G2, G3/G4; race-white). To establish the correlations between clinico-pathological characteristics and risk score, the 82 samples with clinical information (stage, age, gender, grade, race and TMN) in the in TCGA-PAAD training set were extracted and compared between subgroups in each clinico-pathological characteristics. The Wilcoxon test was used for comparisons between groups while the Kruskal-Wallis test was used for comparisons among groups.

### 2.6 Assessment of independent prognostic value

Univariate and multivariate Cox regression analyses were conducted to establish whether the ZNF-related risk score can be used as an independent predictor of OS for PAAD patients. Stage, age, gender, grade, race, T stage, M stage, N stage and risk score were used as the covariates. Clinical factors with *p* < 0.05 after the two cox analyses were considered to be independent prognostic factors that were used to establish the prognostic model. A prognostic nomogram for assessing the 1-, 3- or 5- year survival probability for PAAD patients was established using the “rms” package (RMS, RRID:SCR_007415) (http://www.rms.org.uk/) (Kandimalla et al., 2020). The C-index and calibration curves of the nomogram were used to calculate the discrimination and calibration between the nomogram predicted value and the true survival.

### 2.7 Analysis of the ZNF gene family signature

To investigate the biological processes that are relevant to the ZNF gene family, first, we determined the correlations between all ZNF family genes and risk scores (Pearson |R| > 0.4). The “corrr” package was used for correlation network construction of the obtained genes, while the “clusterProfiler” package was used for GO and KEGG function enrichment analyses of the correlation genes. To establish the differences in immune cell infiltrations, the proportions of 22 immune cell types in the high- and low-risk groups were calculated using the CIBERSORT algorithm (Chen et al., 2018). The “ggplot2” package was used to draw violin diagrams to present the comparison results. To establish the risk score-associated inflammatory activities, the “correlogram” package was used to investigate the correlations between 7 metagenic clusters (HCK, IgG, Interferon, LCK, MHC-I, MHC-II, and STAT1) and risk scores. Relationships between ZNF-related prognostic genes and immunotherapeutic responses were determined by calculating the differences in tumor mutational load (TMB), neoantigen number, clonal neoantigen number and subclonal neoantigen number between the high and low-risk groups.

### 2.8 Regulatory mechanisms of the prognostic genes

Correlation coefficients between prognostic factor expression levels and their methylation levels were determined using the Pearson’s correlations method. Differential analysis was performed on 178 PAAD samples and 4 normal samples using the “limma” package to obtain differentially expressed miRNAs and lncRNAs. Combined with expression trends of prognostic genes, the competing endogenous RNA (ceRNA) regulatory network was constructed based on the lncRNA-miRNA-mRNA regulatory mechanism.

### 2.9 Validation of prognostic gene expression

In order to verify the expression of the prognostic genes, the TCGA-PAAD cohorts were analyzed and compared with the normal individuals both in the GTEx (with the normalization tool of RLE from DESeq2 R package to obtain normalized count data) and ANTE datasets by the wilcox. test as well as GSE28735 and GSE15471. Immunohistochemical results of prognostic genes in PAAD tissues were searched using Human Protein Atlas (HPA,RRID:SCR_006710) (HPA: https://www.proteinatlas.org/).

### 2.10 Cell RT-qPCR validation

Four strains of pancreatic cancer cells were cultured to establish a group of normal pancreatic epithelial cells as the control group for cytological verification of model genes. Cells were cultured in 10% FBS complete medium to enter the log phase. Then, RNA extraction was performed using a kit (Tiangen, cat#DP430). cDNA synthesis from the extracted RNA was performed using the iScript™ cDNA Synthesis Kit (BIO-RAD, cat#1708891). Cell RT-qPCR validation amplification was performed in a 10 ul system using the iTaq™ universal SYBR^®^ Green SupermixRNA (BIO-RAD, cat#1725121). The experiments were conducted in triplicates, and *p* < 0.05 was set as the threshold for statistical significance (Supplementary Figure S2). The PANC-1, RRID: KCB 200809YJ, BxPC-3, RRID: KCB 200428YJ, SW 1990, RRID: KCB 2012113YJ cells, were acquired from the Kunming Institute of Zoology, Chinese Academy of Sciences. The ASPC-1, RRID: TCHu 8, cells were procured from the cell bank of the Chinese Academy of Sciences while. HPDE6-C7, RRID: BFN60807571 cells were obtained from, Qingqi (Shanghai) Biotechnology Development Co. Ltd.

## 3 Results

### 3.1 Identification of DE-ZNFs and functional analysis in PAAD

**FIGURE 1:**
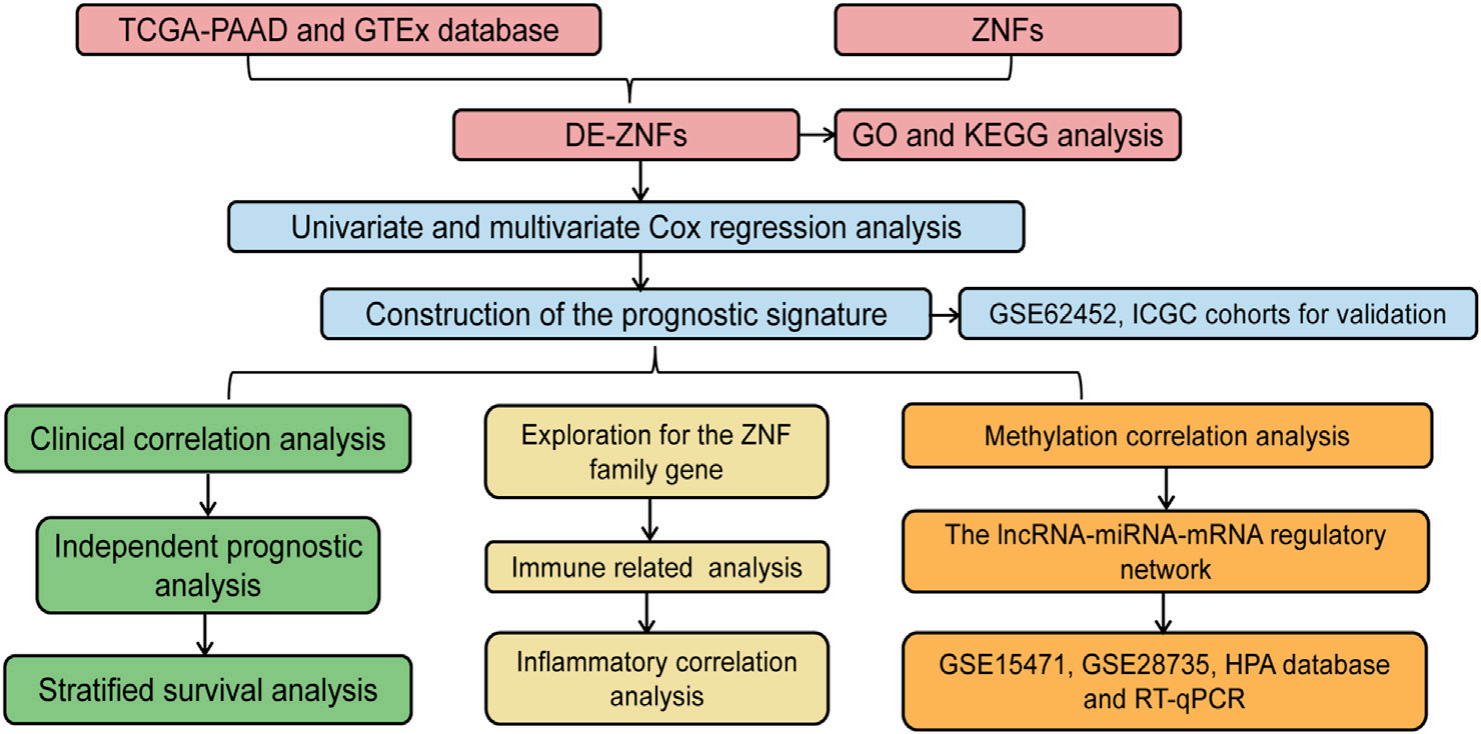
Workflow diagram of this paper.

**FIGURE 2:**
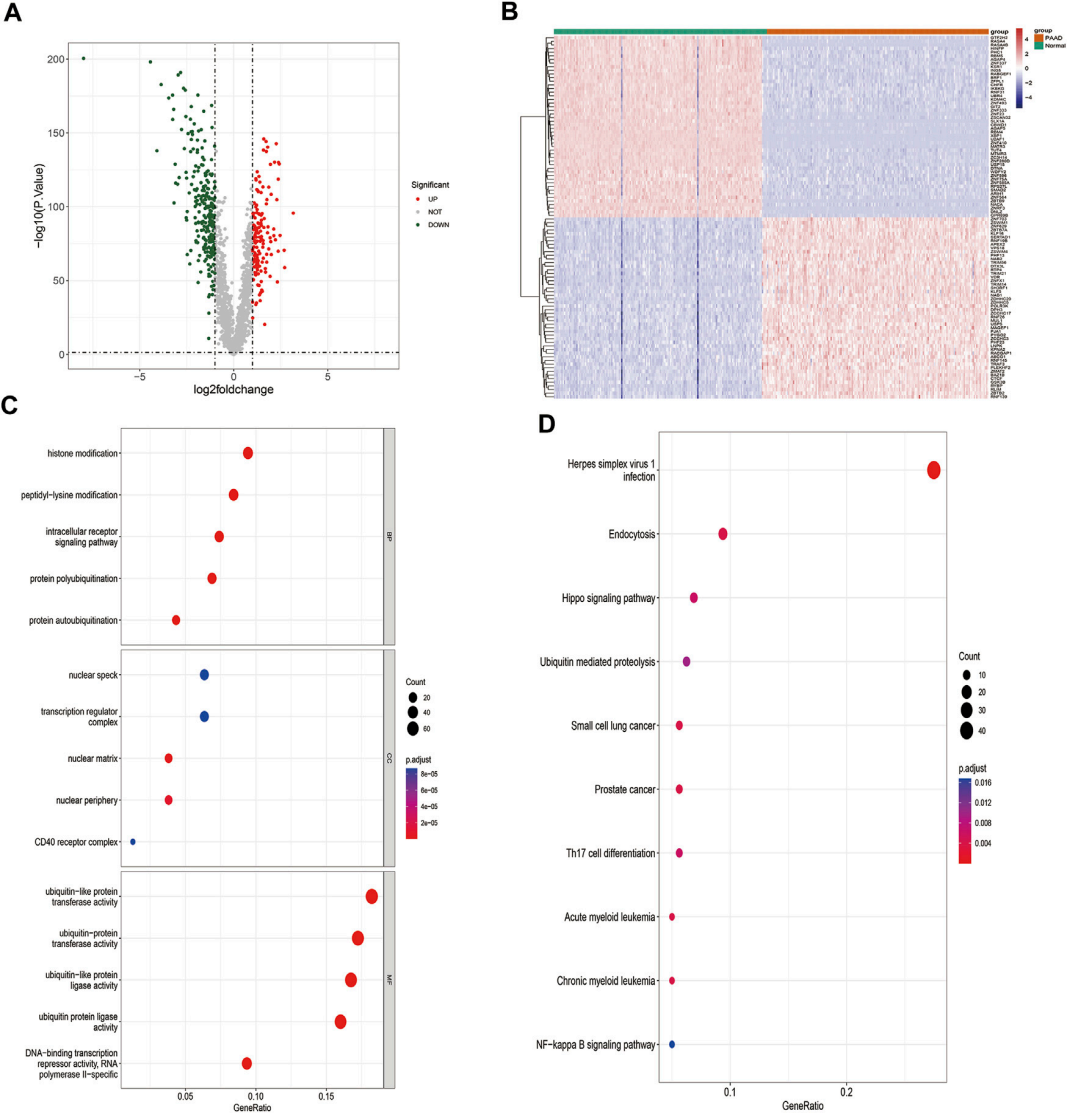
Identification and functional analysis of DE-ZNFs. **(A)**. Volcano plot of differentially expressed genes in PAAD-vs-Normal comparison group **(B)**. Heat map of differentially expressed genes, 150 upregulated, 257 downregulated, |log2 (fold change)|>1 and *p* < 0.05 **(C)**. Top 5 GO BP, CC, and MF enrichment results of DE-ZNFs **(D)**. Top 10 enriched KEGG pathways of DE-ZNFs.

The workflow for this study is shown in Figure 1. A total of 407 DE-ZNFs (150 were upregulated and 257 were downregulated) were identified between the PAAD and normal control tissue samples (Figures 2A, B). The 407 DE-ZNFs were found to be enriched in 226 BPs, 10 CCs, 61 MFs, and 18 KEGG signaling pathways. These included biological processes such as protein autoubiquitination, intracellular receptor signaling pathway, protein polyubiquitination (Figure 2C), and signaling pathways such as herpes simplex virus 1 infection, Th17 cell differentiation, and NF-kappa signaling pathway (Figure 2D).

### 3.2 Construction of the prognostic model for PAAD

**FIGURE 3:**
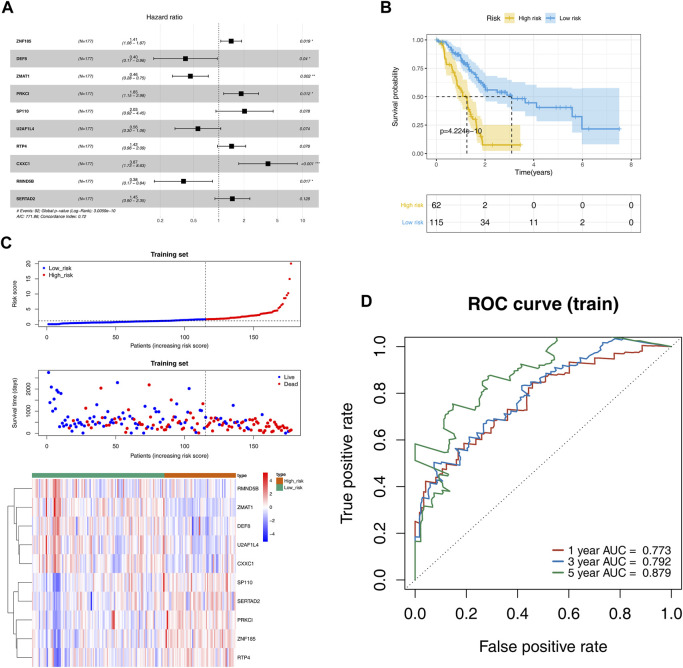
Evaluation and validation of the prognostic risk models. **(A)**. Cox regression analysis forest plot shows that 10 DE-ZNFs were used as parameters to construct the best prognosticis model **(B)**. OS survival curves showing thes survival probabilities of high and low risk groups **(C)**. The scatter plot of the risk score and survival time as well as heatmap of gene expression for each PAAD sample in high and low risk groups, which were sorted from left to right according to the risk score **(D)**. ROC curves of the prognostic model at the 1-, 3-, and 5-year time nodes.

Univariate Cox regression analysis revealed 36 DE-ZNFs that were significantly associated with OS (Supplementary Figure S3). The significantly expressed genes were subjected to multivariate Cox regression analysis to construct the prognostic model. The forest map was used for visualization (Figure 3A). The prognostic model exhibited the best performance when 10 DE-ZNFs were included. The risk score for each sample was calculated based on expression levels of the 10 prognostic genes. Risk score = ZNF185* 0.340812 + DEF8 * −0.91561 + ZMAT1 *-0.77978 + PRKCI * 0.6138 + SP110* 0.70712+U2AF1L4 * −0.57379 + RTP4 * 0.351366 + CXXC1 *1.352264 + RMND5B * −0.96994 + SERTAD2 * 0.373257. Based on the median risk score, PAAD samples were divided into high and low-risk groups. The Kaplan–Meier curve revealed that samples in the high-risk group exhibited worse OS outcomes than those in the low-risk group (Figure 3B). The risk curve and scatter plot were generated to show the risk score and survival status for each PAAD sample. The risk coefficient and mortality in the high-risk group were higher than those in the low-risk group (Figure 3C). A heat map of the 10 prognostic gene expression profiles in PAAD samples revealed that DEF8, RMND5B, CXXC1, ZMAT1, and U2AF1L4 were highly expressed in the low-risk group, while RTP4, ZNF185, PRKCI, SERTAD2 and SP110 were highly expressed in the high-risk group (Figure 3D). The ROC curves were plotted at time nodes of 1, 3, and 5 years. The AUCs of the ROC curves were all greater than 0.7, indicating a good efficacy of the prognostic model (Figure 3E). Validation of the prognostic model was performed using the GSE62452 and ICGC datasets, and the results were comparable to those of the training set (Supplementary Figure S4).

### 3.3 Risk score performance

**FIGURE 4:**
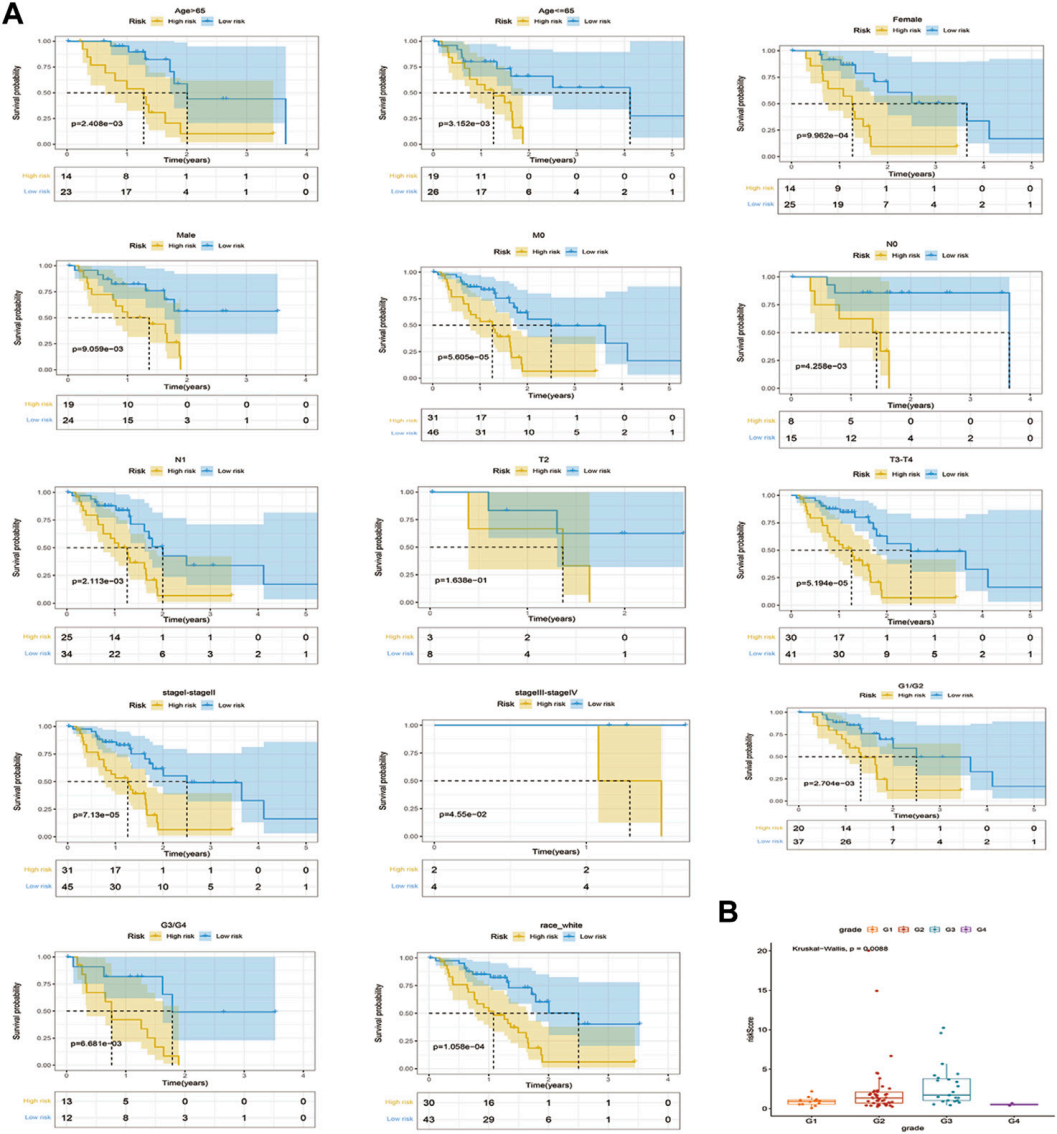
Stratified survival analysis of risk scores and correlation analysis of clinicopathological characteristics. **(A)**. K-M curves of PAAD patients in high and low risk groups of Age > 65, Age <= 65, female, male, M0, T2, T3-T4, stageI-stage II, stage III-stageIⅤ, Race white, G1/G2, and G3/G4 **(B)**. Correlations between stages of grade and risk models.

After stratifying the clinical characteristics, there were significant differences in risk scores between the high and low risk groups in Age>65, Age<=65, female, male; M0, T3-T4, stage I-stage II, stage III-stage IV, G1/G2, G3/G4 and race-white (Figure 4A). Correlation analysis of clinico-pathological factors such as stage, age, gender, grade, race, and TMN with prognostic models for the 82 TCGA - PAAD samples revealed significant differences in risk scores only in grade Figure 4B; Supplementary Figure S5.

### 3.4 Independent prognostic factors in PAAD

**FIGURE 5:**
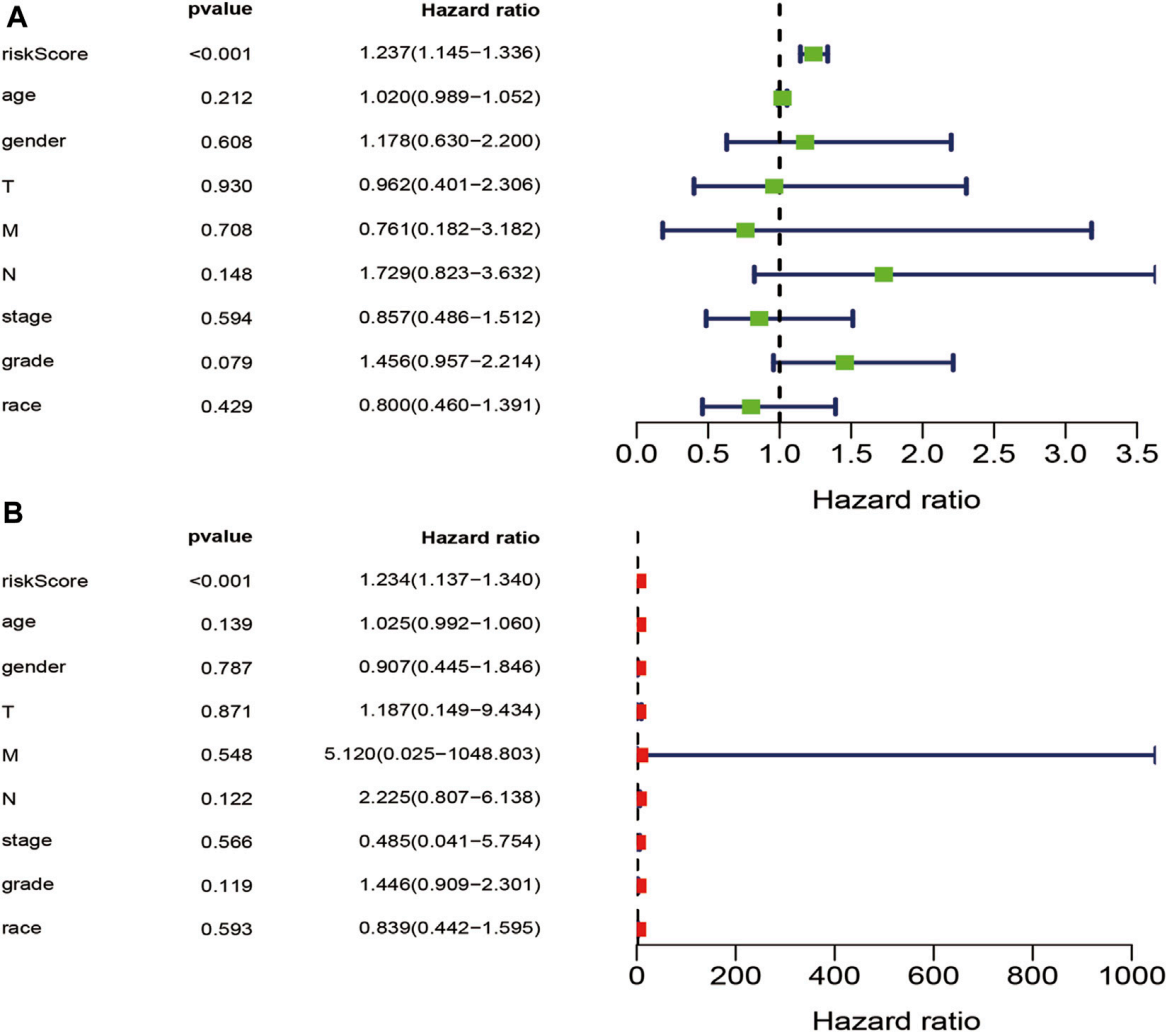
Univariate and multivariate independent prognostic analysis. **(A)**. Univariate Cox independent prognostic analysis of stage, age, gender, grade, race, T stage, M stage, N stage, and riskScore **(B)**. Multivariate Cox independent prognostic analysis of stage, age, gender, grade, race, T stage, M stage, N stage, and riskScore.

Clinico-pathological factors, such as stage, age, gender, grade, race, TMN, and risk score were subjected to univariate and multivariate Cox regression analyses to establish the independent prognostic factors for PAAD. The risk score was found to be a significant prognostic factor in both Cox analyses (*p* ≤ 0.05), suggesting that the risk score was is an independent prognostic factor for PAAD patients (Figures 5A, B).

### 3.5 Construction of the nomogram

**FIGURE 6:**
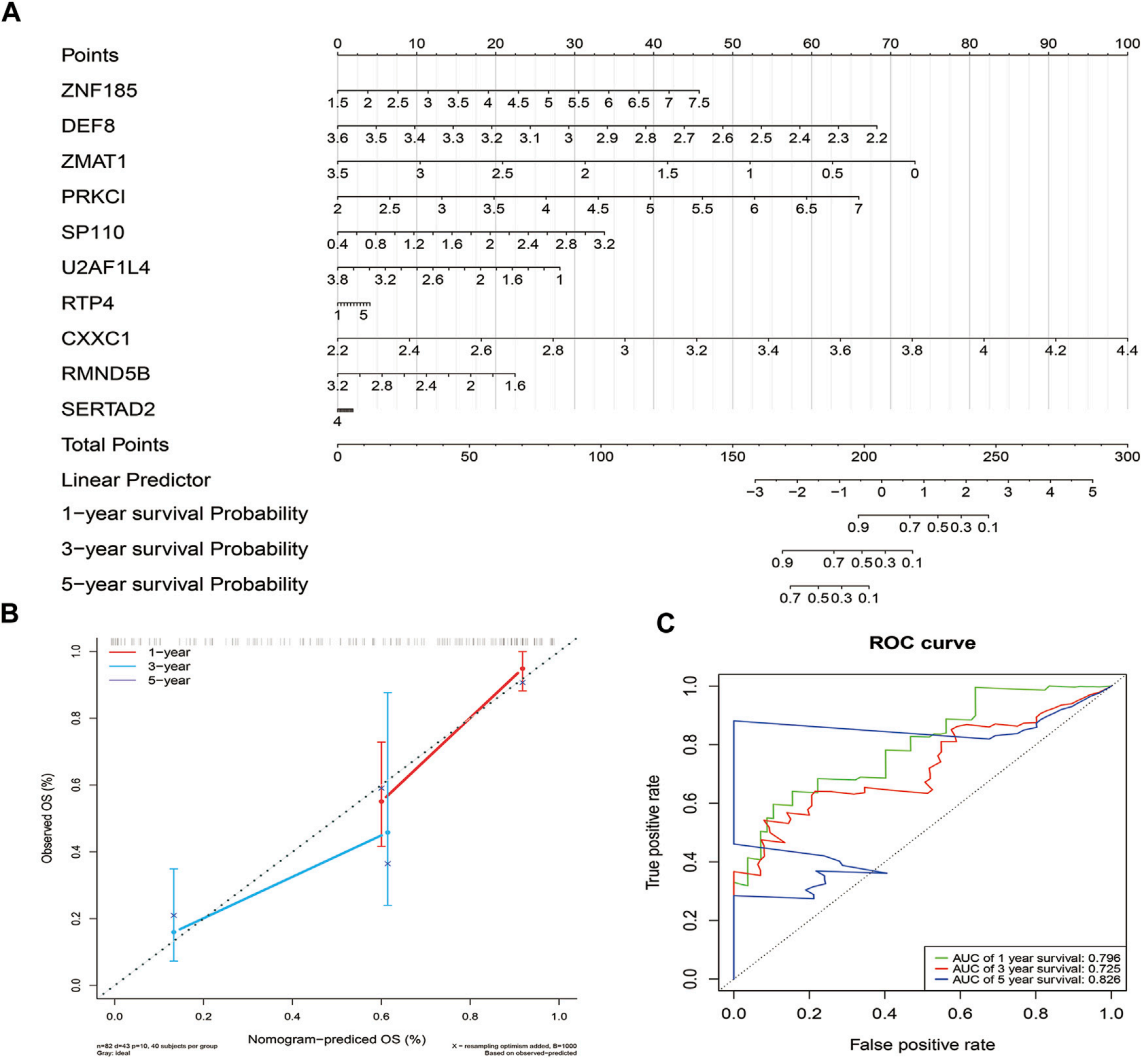
Construction and validation of the nomogram. **(A)**. The nomogram based on the 10 prognostic genes of the risk score **(B)**. Calibration curve of the nomogram.The diagonal dotted line slope is 1. **(C)** ROC curves of the nomogram.

The independent prognostic factors were used to establish a nomogram for prediction of 1-, 3- and 5-year OS outcomes in TCGA - PAAD cohorts. Ten prognostic genes, including DEF8, RMND5B, CXXC1, ZMAT1, U2AF1L4, RTP4, ZNF185, PRKCI, SERTAD2 and SP110 were included in the model (Figure 6A). The points of the factors indicate their corresponding contribution to the survival probability. The actual OS and nomogram-predicted OS outcomes at 1 and 3 years matched well, as shown by the calibration curves (Only 1 patient survived for 5 years, thus, the calibration curve for 5 years was not shown.) (Figure 6B). The AUCs at 1-, 3- and 5- years time nodes were 0.796, 0.725 and 0.826, respectively, revealing that the predictive ability of the nomogram was accurate (Figure 6C).

### 3.6 Biological processes of ZNF family gene signatures

**FIGURE 7:**
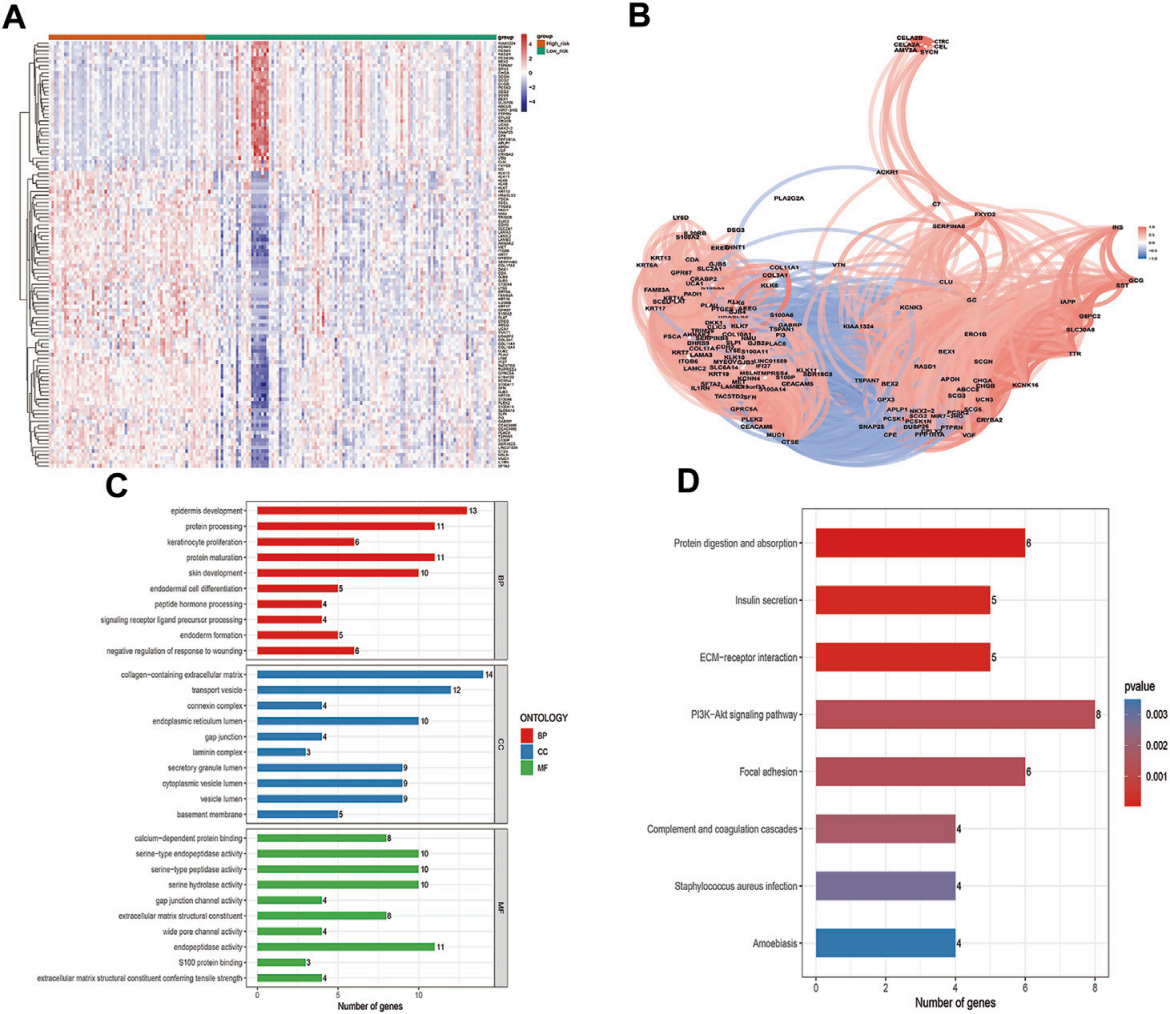
Biological processes involved in ZNF family gene signaling. **(A)**. Heatmap of 115 genes that were closely related to the risk score **(B)**. Correlation network of 115 genes that were closely related to the risk score **(C)**. GO enrichment analysis results of 115 genes that were closely related to the risk score, top 10 BP, CC and MF enriched terms **(D)**. Eight enriched KEGG pathways in which the115 genes that were closely related to risk score were enriched.

The 115 ZNF family signatures that were closely associated with risk scores were identified by correlation analyses (Figure 7A). There were positive correlations between most of the ZNF family signatures and the risk scores (Figure 7B). The 115 ZNF family signatures were enriched in 56 BP, 28 CC, 16 MF, and 8 KEGG signaling pathways, which were significantly associated with epidermis development, protein processing, keratinocyte proliferation biological functions and protein digestion and absorption, insulin secretion, as well as ECM-receptor interaction signaling pathways (Figures 7C, D).

### 3.7 Immune cell infiltration landscape

**FIGURE 8:**
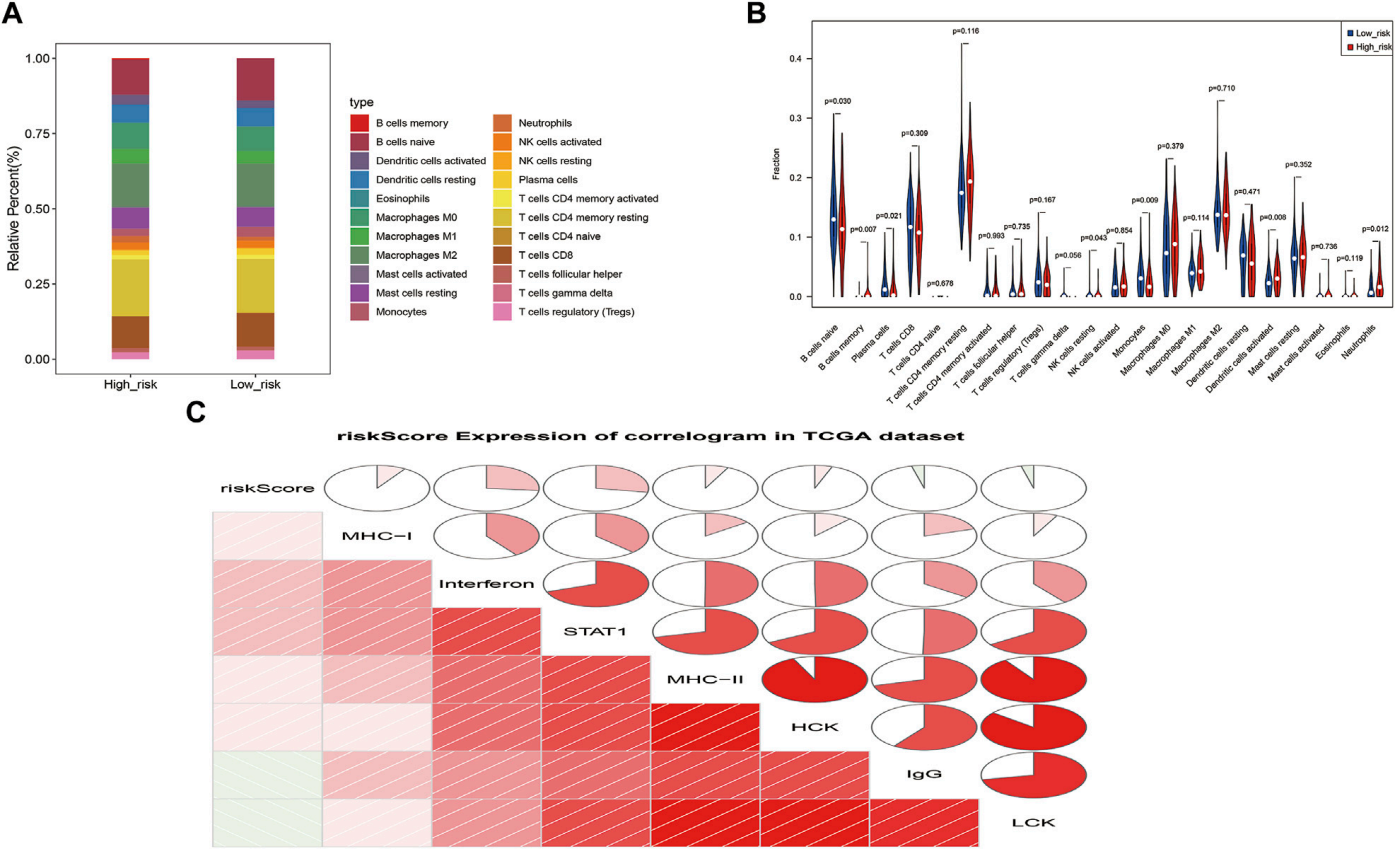
Correlation analysis of ZNF family gene signaling with cellular immunity and inflammation. **(A)**. Heat map of the proportions of 22 immune cells in high and low risk groups **(B)**. Violin plot of the infiltration abundance of 22 immune cells in the high and low risk groups **(C)**. Correlation plot of the risk score and seven metagene clusters.

The proportions of 7 immune cells (naïve B cells, memory B cells, plasma cells, resting NK cells, monocytes, activated dendritic cells and neutrophils) differed between the risk groups (*p* < 0.05; Figures 8A, B). These results reveal a dysregulated tumor immune microenvironment. Correlation analysis of seven meta genic clusters (HCK, IgG, interferons, LCK, MHC-I, MHC-II, and STAT1) with risk scores showed that the risk score was negatively correlated with IgG and LCK and weakly positively correlated with HCK, interferons, MHC-I, MHC-II, and STAT1 (Figure 8C).

### 3.8 Relationships between immunotherapy and ZNF family gene signatures

**FIGURE 9:**
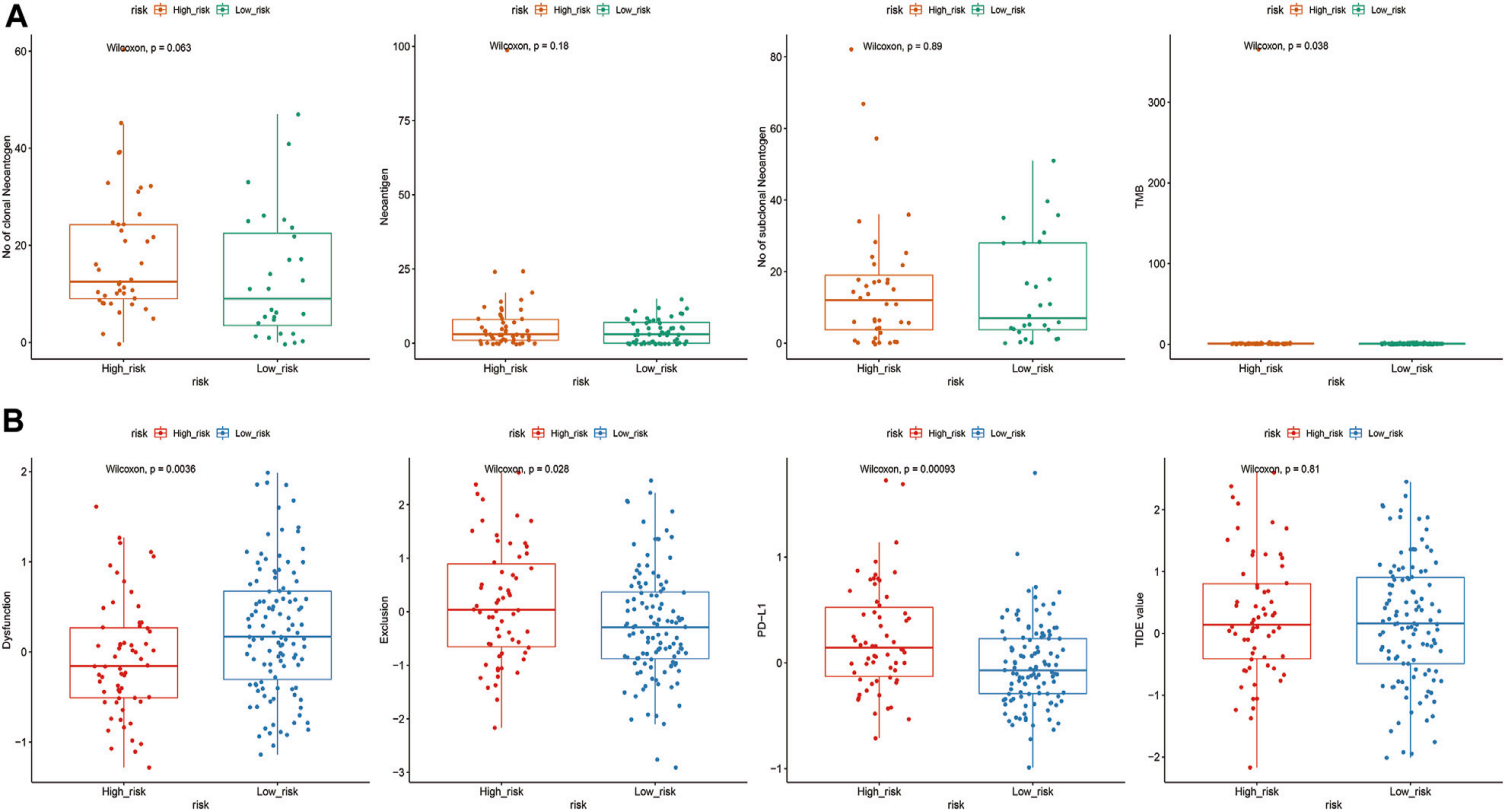
Correlation analysis of ZNF family gene risk signals and immunotherapy. **(A)**. Differences in abundance of TMB, neoantigens, cloned neoantigens and subcloned neoantigens between high and low risk groups **(B)**. Expressions of TIDE, Dysfunction, Exclusion and PD-L1 in high and low risk groups.

Relationships between ZNF family signatures and immunotherapeutic responses were analyzed. The number of TMB was significantly high in the low risk group, compared to the high risk groups (*p* < 0.05; Figure 9A). Exclusion and PD-L1 were significantly differentially expressed between the high and low risk groups (*p* < 0.05), while TIDE was not significantly differentially expressed between the groups (Figure 9B).

### 3.9 Regulatory mechanisms of prognostic genes

**FIGURE 10:**
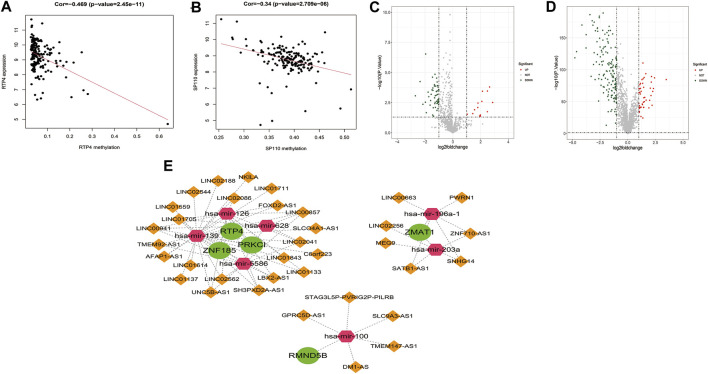
Regulatory mechanisms of risk model genes. **(A)**. Scatter plot of the correlation between risk model genes RTP4 and their methylation levels **(B)**. Scatter plot of the correlation between risk model genes SP110 and their methylation levels. **(C)**. Volcano plot of differentially expressed miRNAs in PAAD-vs-Normal comparison group **(D)**. Volcano plot of differentially expressed lncRNAs in PAAD-vs- Normal comparison group **(E)**. The ceRNA regulatory network with 5 risk model genes, 7 miRNAs and 35 lncRNAs. The green circles represent the risk model genes, the pink hexagons represent miRNAs, and the orange diamonds represent lncRNAs.

Analysis of correlations between prognostic genes and their methylation levels arevealed significant negative correlations between RTP4 and SP110 and their methylation levels (Figures 10A, B; Supplementary Figure S6). There were 66 differentially expressed miRNAs between the 178 PAAD samples and 4 normal samples, of which 14 were upregulated while, 52 were downregulated (Figure 10C). Moreover, there were; and 199 differentially expressed lncRNAs, of which 49 were upregulated while 150 were downregulated (Figure 10D). Among the 10 prognostic genes, ZNF185, PRKCI, RTP4, and SERTAD2 were upregulated while DEF8, ZMAT1, SP110, U2AF1L4, CXXC1, and RMND5B were downregulated. We sequentially extracted three expression matrices from mRNA/miRNA/lncRNA. The expression data for 10 prognostic genes (up-4, down-6), differentially expressed miRNAs (up-14, down-52) and differentially expressed lncRNA (up-49, down-150) were extracted. Finally, based on potential regulatory relationships in mRNA/miRNA/lncRNA, we constructed a ceRNA network consisting of 5 prognostic, 7 miRNAs and 35 lncRNAs (Figure 10E).

### 3.10 Expressions of the prognostic genes

**FIGURE 11:**
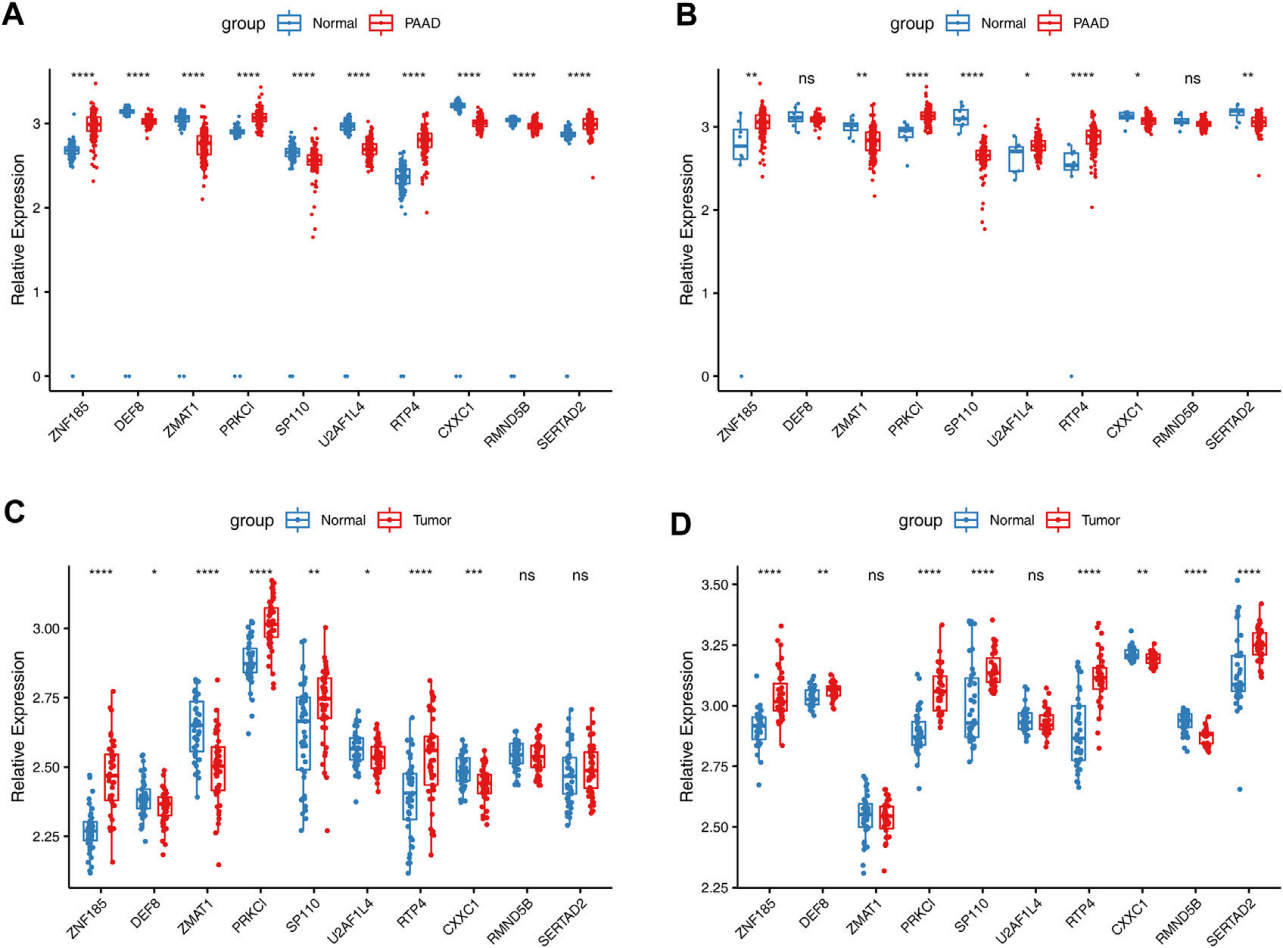
Validation of expressions of risk model genes in TCGA and GEO datasets. **(A)**. The expressions of 10 risk genes between PAAD TCGA-PAAD and normal samples GTEx-normal cohorts in TCGA **(B)**. The expressions of 10 risk genes between TCGA-PAAD and ANTE-normal cohorts. The expressions of 10 risk genes in GSE28735 **(C)**. and GSE15471 **(D)** datasets.

The expression levels of ten prognostic genes (ZNF185, PRKCI, RTP4, SERTAD2, DEF8, ZMAT1, SP110, U2AF1L4, CXXC1, and RMND5B) were examined in the datasets TCGA, GSE28735, and GSE15471. The results between the TCGA-PAAD and GTEx-normal data after normalization exhibited that among these genes expression was significantly different between the two groups (Figure 11A). Similarly, the expressions of eight risk genes between TCGA-PAAD and ANTE-normal cohorts had apparent differences except for DEF8 and RMND5B (Figure 11B). However, it was noticed that the expression trends of SERTAD2 and U2AF1L4 were opposite in two cohorts above. For the two GEO datasets, it was showed that the expression patterns of various prognostic genes were similar to those in the TCGA cohort except for SERTAD2 and U2AF1L4 as well. Furthermore, it was noteworthy that the SP110 expression was upregulated in PAAD samples compared to controls, while it expressed higher in GTEx-normal and ANTE-normal samples (Figures 11C, D).

**FIGURE 12:**
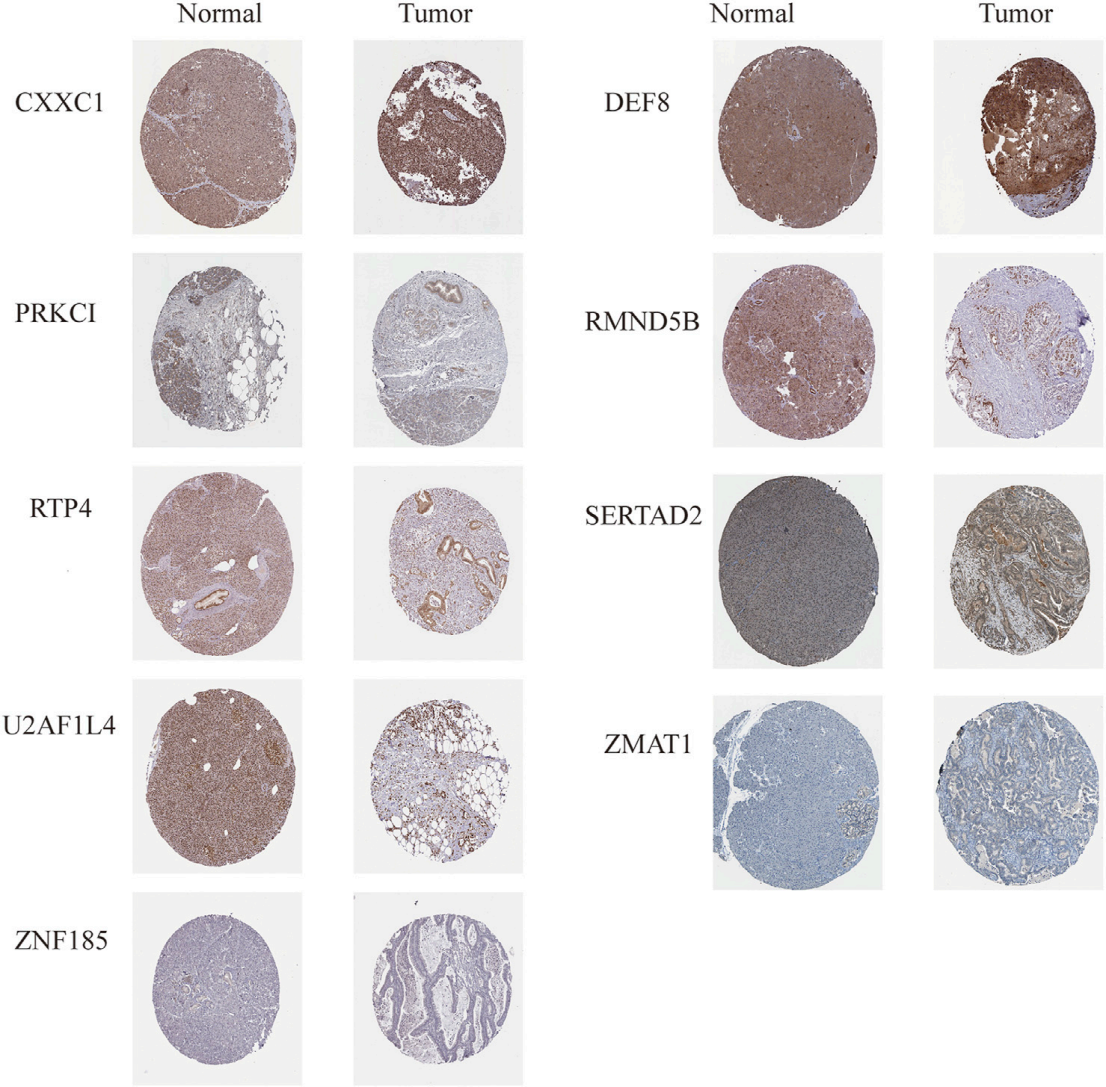
Expression levels of 9 risk model genes were validated in HPA database.

Analysis of protein levels of prognostic genes in PAAD and normal tissues in the HPA database further revealed that SERTAD2 (in Cytoplasmic, membranous) proteins were expressed at significantly higher levels in tumor tissues than in normal controls, confirming the expression results in (Figures 11A, D rather than that in (Figures 11B,C; While the expression of U2AF1L4 proteins (in Nuclear) was not significantly different (Figure 12), and no immunohistochemical result was available for SP110 protein in the HPA database, indicating that the expression patterns of U2AF1L4 and SP110 remained to be further studied. Besides the expression levels of PRKCI (in Cytoplasmic, membranous), ZMAT1 (in Cytoplasmic, membranous), CXXC1 (in Nuclear), DEF8 (in Cytoplasmic, membranous), RTP4 (in Cytoplasmic, membranous), RMND5B (in Nuclear), and ZNF185 were confirmed and in accordance with the results of public datasets.

### 3.11 PADD cell validation

**FIGURE 13:**
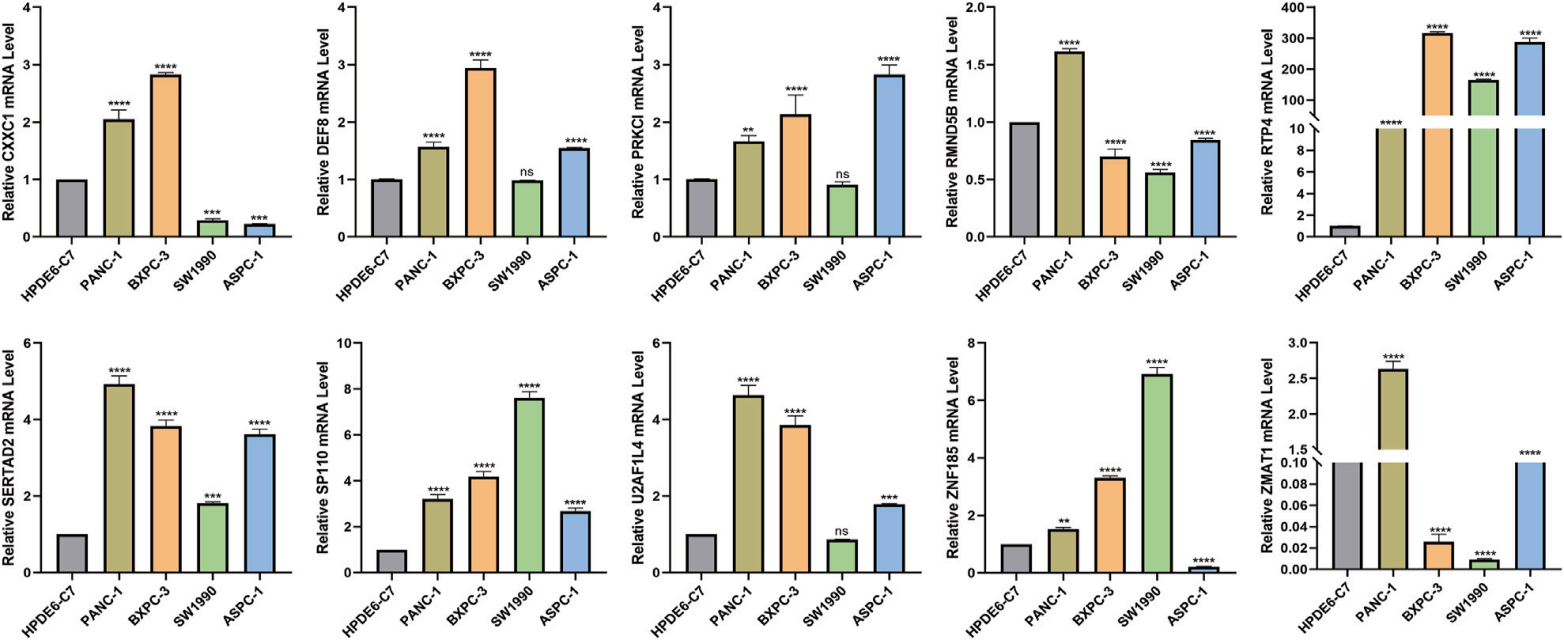
Validation of expression levels of 10 risk model genes in pancreatic cancer cells by Cell RT-qPCR validation * represents *p* < 0.05, **, represents *p* < 0.01, *** represents *p* < 0.001, and **** represents *p* < 0.0001.

The HPDE6-C7 cell line was used as the control group, and pancreatic carcinoma *in situ* and metastatic cancer cell lines were used as the experimental group to investigated the expression of 10 risk model genes, where the expression levels in the control group were basically the same. It could be seen that RTP4, SERTAD2, and SP110 were all highly expressed in the four pancreatic cancer cell lines, which were in concordance with publicly available GEO data, suggesting that they are closely related to the occurrence and metastasis of the pancreas at the cellular level. The expression of DEF8, RRKCI, U2AF1L4 were distinctly higher in PANC-1, BXPC-3 and Aspc-1 metastatic cell lines. Moreover, the expression levels of the ZNF185, CXXC1, RMND5B, and ZMAT1 genes changed significantly with different cell lines (Figure 13).

## 4 Discussions

Pancreatic cancer is a highly invasive malignant tumor, that invades many organs, including the stomach, common bile duct, duodenum, superior mesenteric vein, and celiac artery in the terminal stages. Extensive lymphatic metastasis is often accompanied by nerve sheath metastasis, which results in extremely high fatality rates. Pancreatic cancer development involves complex biological processes. It is closely associated with cell phenotypes that are related to autophagy, histone methylation, hypoxia tolerance, and apoptosis among others and is crucial to improve the treatment plans and prognostic outcomes of PAAD (Gupta et al., 2021; Liu et al., 2021; Wang Y. et al., 2021). Studies on correlations of biological targets for early pancreatic cancer diagnosis are still in early stages. In this study, survival and RNA-seq data for PAAD patients from TCGA and GSE databases were downloaded, and the differentially expressed genes and ZNF data obtained and analyzed to construct a prognostic risk model. The prognostic risk model including 10 independent risk factors, such as ZMAT1, PRKCI, ZNF185, SERTAD2, CXCC1, U2AF1L4, RTP4, SP110, and DEF8, was established and validated in GEO and ANTE datasets. The q-PCR analysis showed that the Panc-1 cell line, derived from human ductal cell carcinoma and the pancreatic adenocarcinoma Bxpc-3 basically showed a higher expression level, while the expression in the metastatic carcinoma cells of pancreatic cancer was different. It was found that RTP4, SERTAD2, and SP110 were significantly expressed in all pancreatic cancer cells, including those of pancreatic ductal carcinoma, pancreatic adenocarcinoma and metastatic carcinoma, providing a reliable basis for subsequent elucidation of pancreatic cancer pathogenesis.

It has been reported that ZMAT1 induces p21 expressions via the SIRT3-p53 signaling pathway to inhibit pancreatic cancer cell proliferation and induce S/G2 cell cycle arrest (Ma et al., 2022). PRKCI-mediated ablation of pancreatic acinar cells resulted in p62 aggregation and loss of autophagic vesicles. Pancreatic PRKCI knockdown significantly increased pancreatic immune cell infiltrations acinar cell DNA damage, apoptosis, and promoted KrasG12D mediated pancreatic intraepithelial neoplasia, promoting tumor growth (Inman et al., 2022). Pancreatic cancer chemotherapy tolerance is associated with poor survival and prognostic outcomes of patients. Pancreatic cancer HEAT repeat-containing protein 1 (HEATR1) deficiency can affect pancreatic cancer chemotherapy sensitization via the upregulation of ZNF185 (Fang Y. et al., 2020). In the prognostic risk model of this study, ZMAT1 is a low-risk gene for pancreatic cancer, while PRKCI and ZNF185 are high-risk genes for pancreatic cancer.

A part from the above three genes, the other independent risk factors have not been reported to be pancreatic cancer-related. However, basic experiments and high-throughput data analysis of other solid tumors confirmed that the other risk model genes are involved in tumor pathogenesis. This evidence provides the author with more reliable information and enthusiasm for exploration. Elucidation of the importance of zinc finger proteins in pancreatic cancer mayinform on novel approaches for identification of treatment targets and overcoming chemotherapeutic resistance among others. As transcriptional regulators of the largest mammalian system, zinc finger proteins are involved in regulation of tumor mechanisms via multiple pathways. The central member of the family of TLS polymerases (REV1) upregulates SERTAD2 expressions in a Rad18-dependent manner, thereby enhancing lung cancer development (Chen et al., 2022). Wei Wang et al. performed whole-genome sequencing of invasive and *in situ* patients with cutaneous squamous cell carcinoma and found that DEF8 was highly enriched in invasive cutaneous squamous cell carcinoma (Wang et al., 2018). Through bioinformatics and meta-analysis, Shenying Fang et al. identified 3 melanoma risk-related genes, including DEF8, among 330 unique melanoma genes (Fang S. et al., 2020). In bioinformatics studies of other related tumors, it was found that U2AF1L4 a prognostic factor for renal cancer, especially renal clear cell carcinoma (Wang B. et al., 2021). Kuo-Wei Chang et al. performed whole-exome sequencing of a p53-deficient murine oral cancer cell line and found that SP110 exhibited comparable mutations to those in chemical carcinogenesis-related tongue cancer cell lines in the human TCGA database (Chang et al., 2020).

We intersected the PAAD-related differentially expressed genes in the TCGA database with DE-ZNF to obtain 407 ZNF-related functional genes. The GO and KEGG enrichment analysis revealed that the functional genes were enriched in 226 BP, 10 CC, 61 MF and 18 KEGG signaling pathways. The main enriched related biological signals and pathways were: protein autoubiquitination, intracellular receptor signaling pathway, Th17 cell differentiation, HSV1 infection, and NF-κB signaling pathway. These results show that the functional genes are involved in tumor proliferation, apoptosis, cycle, metastasis, and tumor immunity. Correlation analysis of the ZNF gene family and PAAD showed that the DE-ZNF functional gene risk score was significantly correlation from various tumor immune infiltrating cells and inflammatory cells. In the high-risk group, NK cells, dendritic cells, neutrophils, STAT family, MHC-I, and MHC-II were positively correlated with PAAD pathogenesis, and the immunotherapy target PDL-1 was also significantly different. The α-Enolase (ENO1) specific Th17 cells have specific anticancer effects in PAAD patients, and compared with healthy mucosa, the abundance of Th17 in peripheral blood of tumor patients is low, while the proportion of FOXP3+Tregs is high. The FOXP3+RORγt + Tregs secrete both Th17 and Th2-related pro-inflammatory cytokines, corresponding to elevated Th17- and Th2-mediated immune responses in PDAC patients (Amedei et al., 2013; Chellappa et al., 2016). In solid malignant tumors, such as pancreatic cancer, NF-κB is the main regulatory signaling pathway that promotes malignancy and chemoresistance. Expressions of GPR87 are significantly upregulated in pancreatic cancer and clinical tissues, and activation of the NF-κB signaling pathway promotes pancreatic cancer metastasis (Wang et al., 2017). In enrichment analysis, herpes simplex virus type 1 (HSV-1) infection was specifically proposed. *In vivo* and *in vitro* studies confirmed that pancreatic cancer cells are highly sensitive to HSV1 virus replication, which can be evaluated as an effective treatment scheme. A study on oral squamous cell carcinoma showed that the co-expression gene of zinc finger protein 71 (ZNF71) was mainly enriched in the HSV1 infection pathway (Gayral et al., 2015; Jiang et al., 2022). 1-Methyl-D-tryptophan (1-MT) has been shown to significantly reduces the activities of cancer stem cells. A high abundance of CD133 + and PDL-1 expressions in the tumor immune microenvironment, suppresses NF-κβ and Wnt/β-catenin signaling pathways in tumors, and decreases the abundance of intra-tumor Treg cells (Alahdal et al., 2018). Partially mature dendritic cells in peripheral blood of PAAD patients, significantly enhanced the expressions of CD83, CD40, B7H3, PDL-1, CCR6 and CCR7, decreased the expressions of ICOSL and DCIR, and improved the survival and prognostic outcomes of patients (Tjomsland et al., 2010). The 10 risk models that we analyzed have so far not been reported to be related to tumor immunity and NF-κB signaling pathway, which will provide new insights into studies on pancreatic cancer cells and tissue variations as well as functions. In summary, studies on pancreatic cancer tumor immunity and other related biological activities that are important in development of optimal immunotherapeutic approaches are in the initial stage.

Tumor epigenetics is regulated by protein methylation. Studies on tumor epigenetics are key in gene mutation research and targeted therapy. Uncontrolled methylation leads to changes in chromatin structure, and increased protein synthesis mediates infinite pancreatic cancer progression (Wang S. S. et al., 2021). Mishra NK et al. performed differential expression analyses on PAAD tissue data and normal samples from the TCGA database, and found that most differential CpG island (CpG) sites were hypermethylated in PAAD, and promoter methylation as well as 5′UTR were associated with gene expressions, and most of them were negatively correlated, while gene body and 3′UTR-related methylation were positively correlated (Mishra and Guda, 2017). The Ras-mediated cancers utilize the METTL13-eEF1AK55me2 dimethylation axis to increase the translational output, and enhance protein synthesis to promote pancreatic cancer progression. Human Arginine Methyltransferase 1 (PRMT1) overexpression enhances HSP70 binding and BCL2 mRNA stability via elements of the 3′UTR. Increased HSP arginine methylation of HSP70 regulates cell malignancy and is involved in pancreatic cancer drug resistance (Liu et al., 2019; Wang et al., 2020). These findings are in tandem with ours. Knockout of CXCC1, also known as CxxC Finger Protein-1 (Cfp1), affects cytosine methylation and regulation of histone H3K4 on chromatin structure and function. A DNA methyltransferase (DNMT) inhibitor disrupted the DNMT1/CFP1 complex and enhanced mouse glioma chemosensitivity (Cheray et al., 2014). We also found that RPT4 is closely associated with methylation. In previous studies, RTP4 was shown to regulates prostate cancer via methylation and is regarded as a precise target, whose expression levels can be used to independently predict the prognosis of HER2(+) breast cancer (Laurin et al., 2013; Xu et al., 2019).

The carboxy terminus of LisH (CTLH) complex representing RMND5B can promote tumor maintenance and rapid proliferation under extreme conditions and is associated with EMT and, wnt/β-catenin pathway. Overexpressed RMND5B has been shown to inhibit NKX3.1 factor in prostate cancer to suppress its ubiquitination and nuclear levels so as to promote tumor proliferation (Huffman et al., 2019). In eukaryotic cells, including tumor cells, protein degradation is mainly achieved via the ubiquitin-protease degradation system, and ubiquitin ligase E3 is the key dominant factor in this degradation system. During tumor progression, ubiquitin ligase E3inhibits gene induction, suppresses the expression regulation function of the star tumor suppressor gene pP53, and then mediates tumor occurrence and development through the cell cycle or apoptosis. RMND5B has E3 ligase activities and its overexpression in tumors is critical for cancer cell therapy resistance (Tjomsland et al., 2010). In this study, RMND5B was found to be low risk in the risk model, and Cell RT-qPCR validation revealed that its levels were suppressed in multiple pancreatic cancer cells, implying that it may exist as a tumor suppressor gene in pancreatic cancer, which contradicts the findings from other studies. However, the specific regulatory factors have yet to be determined. Abnormalities of the ubiquitin-proteasome system are key in PAAD pathogenesis, and the ubiquitin-proteasome UCHL5 can promote tumor progression and dry expression depending on involvement of the ELK3 protein (Yang et al., 2022). These findings are in tandem with our enrichment analysis results.

Due to its early metastasis, difficult operation and low survival characteristics, pancreatic cancer is a “lethal” cancer. In this study, we identified the PAAD-related DE-ZNF functional genes, and conducted an in-depth analysis of the possible mechanisms of PAAD. We identified ten risk model genes that can be used as independent prognostic factors for PAAD. Epigenetic modifications include methylation, ubiquitination, tumor immune microenvironment, and ceRNA gene regulatory networks. Our findings provide a novel basis for in-depth assessment of immunotherapy and clinical diagnosis of pancreatic cancer.

Finally, it is worth noting that there are differences in gene expression among SERTAD2, U2AF1L4, SP110, *etc.*, in the four online datasets, cell validation, and immunohistochemistry results. A search of relevant literature in the past decade found that SP110 is a special transcription factor of tumor involved in the carcinogenic regulation of breast cancer and ovarian cancer (Korakiti et al., 2020; Rooda et al., 2020). U2AF1L4 was reported to be involved in renal clear cell carcinoma, but there is a lack of corresponding mechanism research (Wang B. et al., 2021). SERTAD2 has been reported as an oncogene in pancreatic cancer (Zhang et al., 2022). This result is consistent with our cell verification, and believes that cell experiments are more reliable. The mechanism exploration of pancreatic cancer is a huge challenge for clinical research. The relevant data from the database provides research direction, but it still needs long-term exploration and large sample size research support to obtain accurate basic data.

## Data Availability

The original contributions presented in the study are included in the article/Supplementary Material, further inquiries can be directed to the corresponding authors.
