# Supplementary material for: The diagnostic significance of the ZNF gene family in pancreatic cancer: a bioinformatics and experimental study
Source: Front Genet. 2023 Jun 16;14:1089023. doi: 10.3389/fgene.2023.1089023 (PMC10311482; doi:10.3389/fgene.2023.1089023)
Supplement: Supplementary file 1 [file DataSheet1.PDF]

frontiers

Supplementary Material

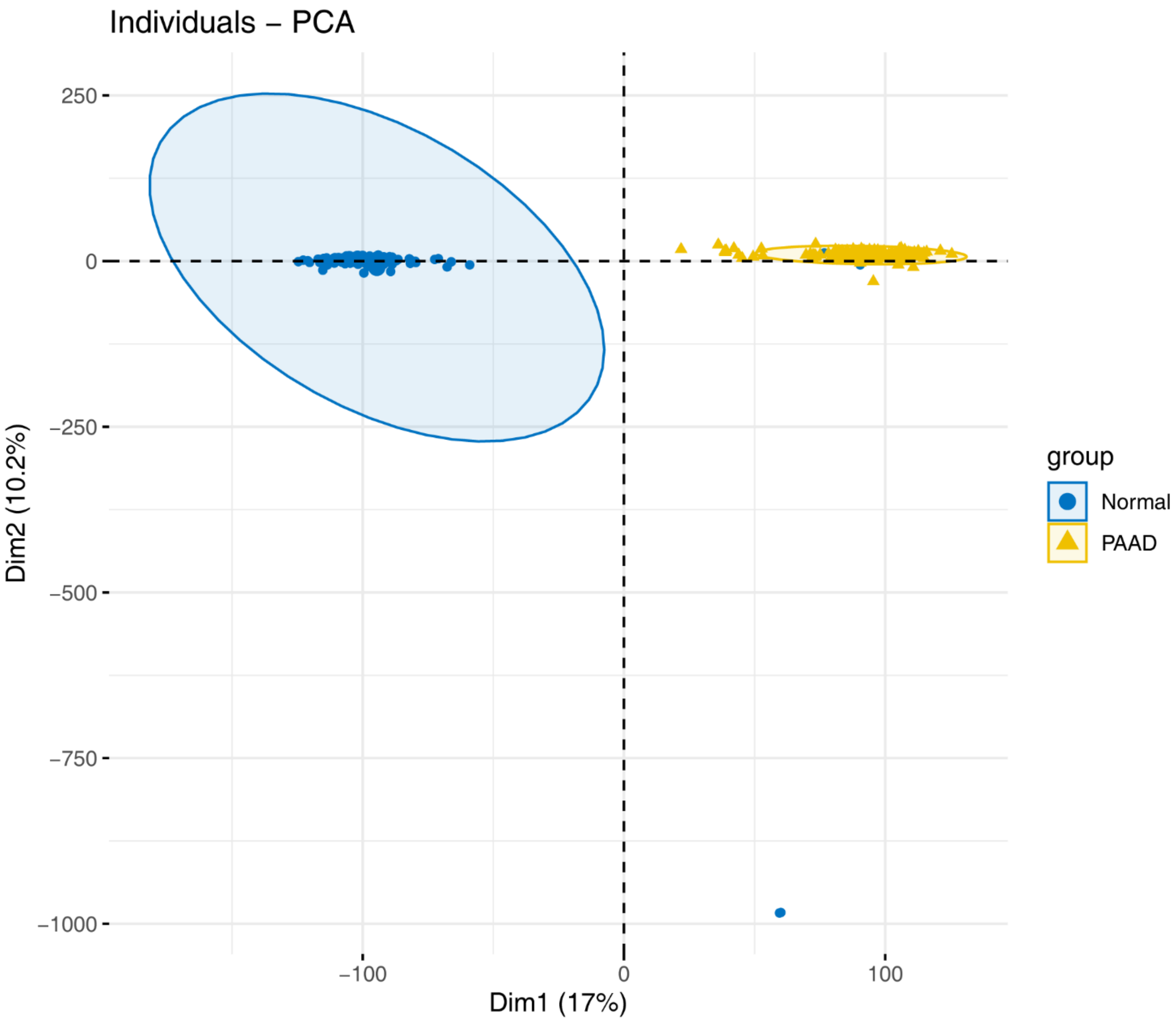

Supplementary Figure 1. PCA profiles of TCGA-PAAD and GTEx normal queues.

**Gene Name**	**Forward Primer**	**Reverse Primer**
CXXC1	GCAAACCGGACATCAACTGC	GCACTCCCGACAGTACCAC
DEF8	AAACATCCGAGTGCTCCTTGA	GTCTGCTTGACGCTCTTGC
PRKCI	GACAACGAACAGCTCTTCACC	CCAGGACGTTCTGGTACACA
RMND5B	AGTTGAATCGAATCCTGGAAGC	GAAGTGCCGAGCATAGCTGA
RTP4	ACATGGACGCTGAAGTTGGAT	TACGTGTGGCACAGAATCTGC
SERTAD2	TGTCCCACATCTACCTCCACA	CTTGAGGACCGTCGAGTTTCT
SP110	CCTATGCCATACACAAGCCATT	CCTCTCCAGTTGGGTGAGAAT
U2AF1L4	TTAGCTTCGATATTCGGGACTGA	TGAATGTCGGCTTGTTGTGAA
ZMAT1	TGTGGAGTGATGCTACAGTTTG	AGGCACTTCATTTTGTTCCCC
ZNF185	TTGGCTGATTATGAGGGGAAGG	TCTCTCGTCTGACAAGTTGCT
GAPDH	GGAGTCCACTGGCGTCTTCA	GTCATGAGTCCTTCCACGATACC

Supplementary Figure 2. Primer sequence design.

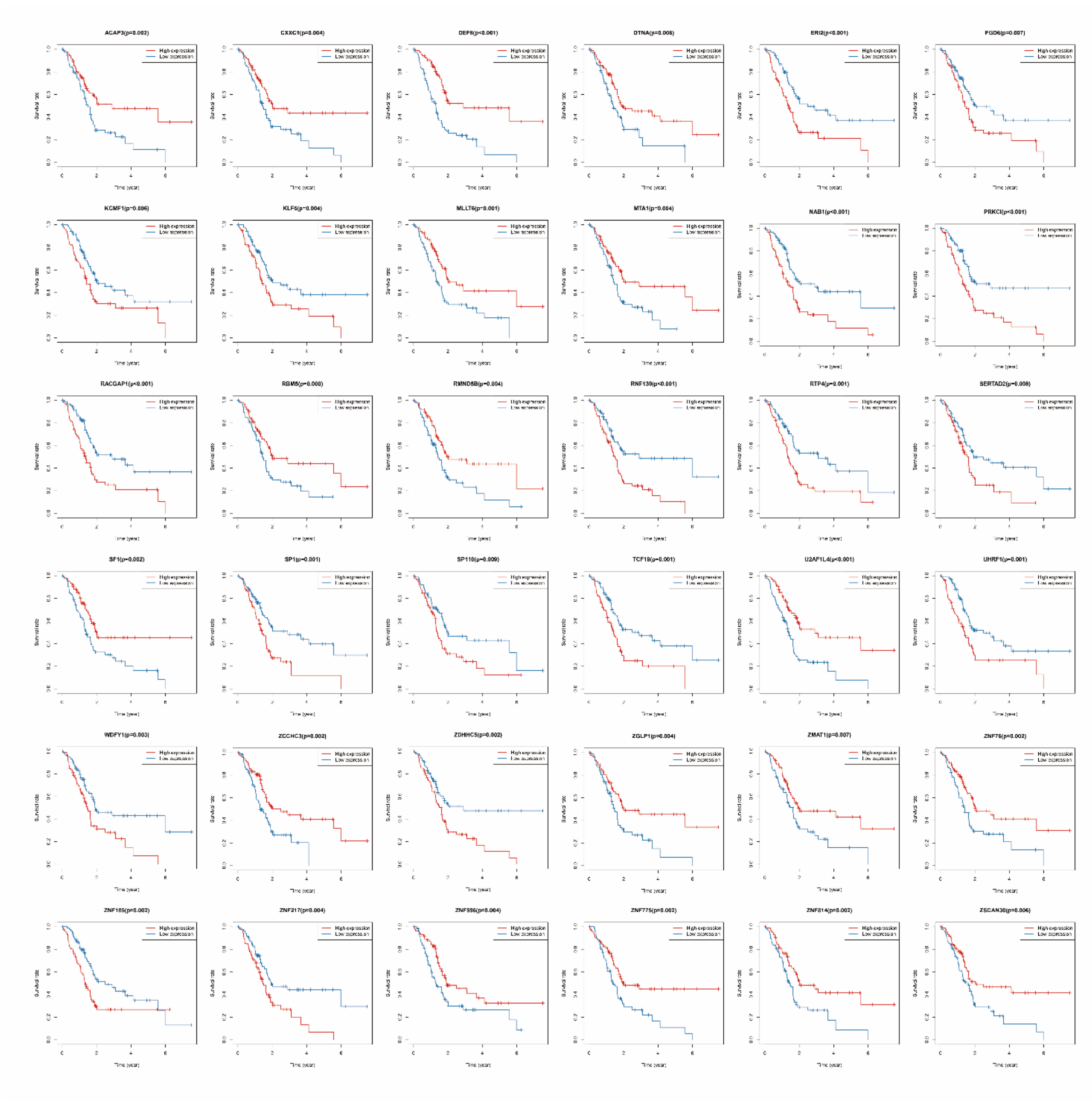

Supplementary Figure 3. Survival curves of 36 genes with univariate Cox and survival analysis p<0.01.

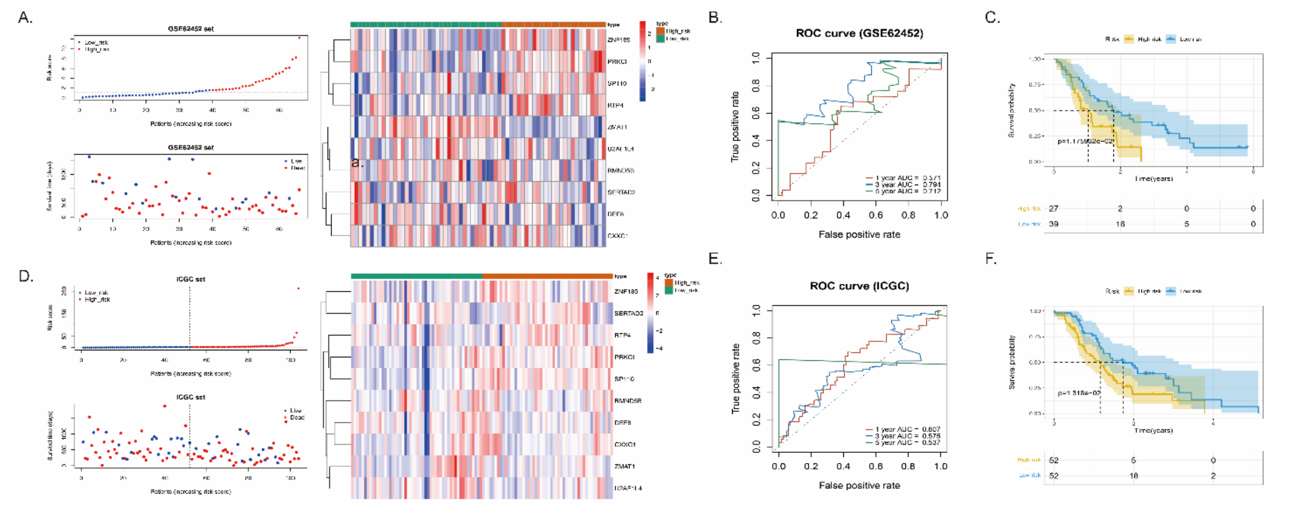

Supplementary Figure 4. Evaluation and validation of prognostic risk models.(A). Risk curve of high and low risk groups in GSE62452. (B). ROC curve of 1/3/5 years in GSE62452 dataset. (C).Survival curve of high-risk groups in GSE62452 dataset. (D). Risk curve of high and low risk groups in ICGC dataset. (E). ROC curve of 1/3/5 years in ICGC dataset. (F). Survival curve of high-risk groups in ICGC.

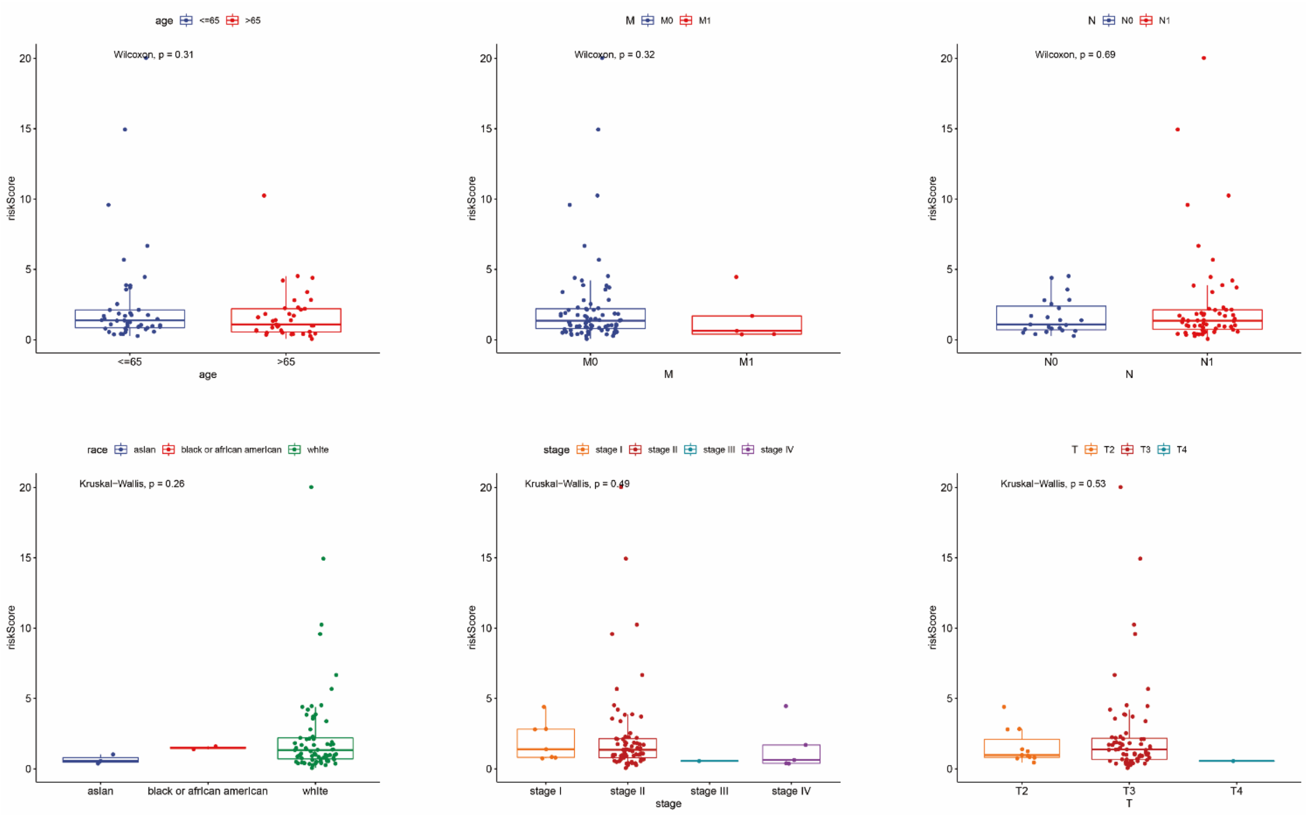

Supplementary Figure 5. Expression of TCGA-PAAD under different clinicopathological characteristics.

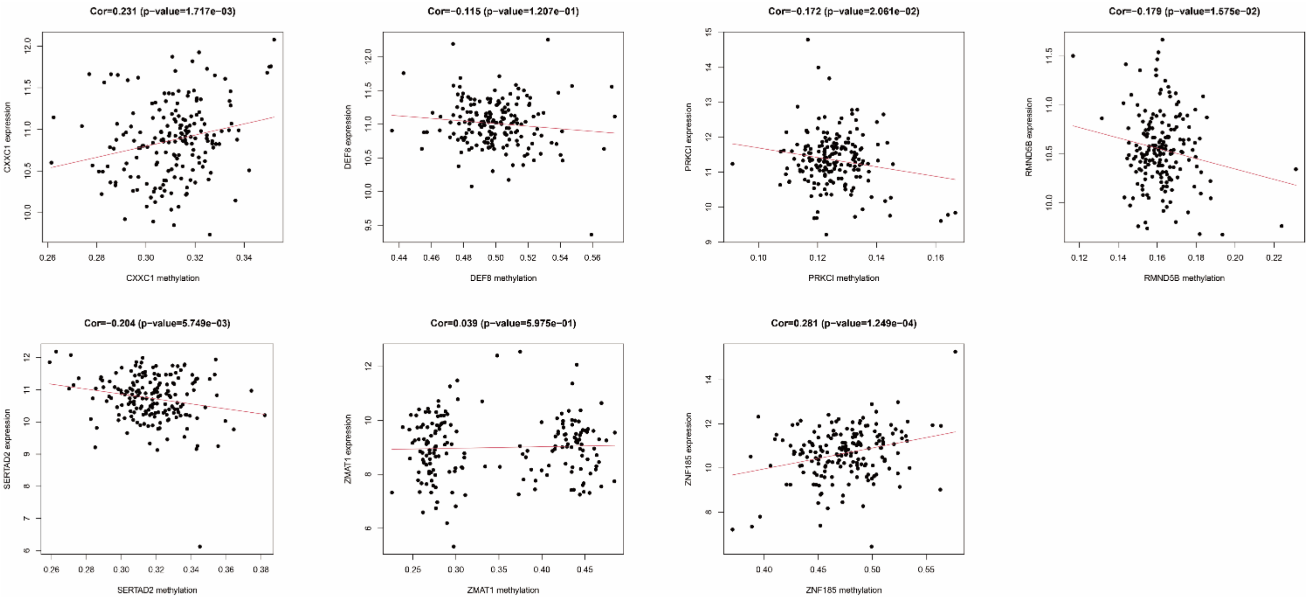

Supplementary Figure 6. Scatter diagram of correlation between risk model gene and its methylation level.
